# Temperature dependence of amorphous magnesium carbonate structure studied by PDF and XAFS analyses

**DOI:** 10.1038/s41598-021-02261-8

**Published:** 2021-11-24

**Authors:** Gen-ichiro Yamamoto, Atsushi Kyono, Satoru Okada

**Affiliations:** Division of Earth Evolution Sciences, Graduate School of Life and Environmental Sciences, University of Tsukuba, 1-1-1 Tennodai, Tsukuba, 305-8572 Japan

**Keywords:** Mineralogy, Nanoscale materials, Structural materials

## Abstract

Mineral trapping through the precipitation of carbonate minerals is a potential approach to reduce CO_2_ accumulation in the atmosphere. The temperature dependence of amorphous magnesium carbonate (AMC), a precursor of crystalline magnesium carbonate hydrates, was investigated using synchrotron X-ray scattering experiments with atomic pair distribution function (PDF) and X-ray absorption fine structure analysis. PDF analysis revealed that there were no substantial structural differences among the AMC samples synthesized at 20, 60, and 80 °C. In addition, the medium-range order of all three AMC samples was very similar to that of hydromagnesite. Stirring in aqueous solution at room temperature caused the AMC sample to hydrate immediately and form a three-dimensional hydrogen-bonding network. Consequently, it crystallized with the long-range structural order of nesquehonite. The Mg K-edge X-ray absorption near-edge structure spectrum of AMC prepared at 20 °C was very similar to that of nesquehonite, implying that the electronic structure and coordination geometry of Mg atoms in AMC synthesized at 20 °C are highly similar to those in nesquehonite. Therefore, the short-range order (coordination environment) around the Mg atoms was slightly modified with temperature, but the medium-range order of AMC remained unchanged between 20 and 80 °C.

## Introduction

Mineral carbonation is a promising approach for reducing the concentration of CO_2_ in the atmosphere^1,2^. There are four main trapping mechanisms that can securely store CO_2_, namely, structural/stratigraphic trapping, residual trapping, solubility trapping, and mineral trapping^3–6^. Mineral trapping involves the injection of CO_2_ into basalt or ultramafic rock, whereby a geochemical reaction occurs between the injected CO_2_ and the alkaline minerals in the basalt or ultramafic rock, leading to the precipitation of carbonate phases. This effectively locks the CO_2_ in immobile secondary phases for geological timescales^6^ and minimizes the risk of leakage to the atmosphere. The inherent stability of mineral carbonation is evidenced by the fact that more than 70% of the total carbon in the Earth’s crust is present in the form of carbonates^7^.

**Figure 1:**
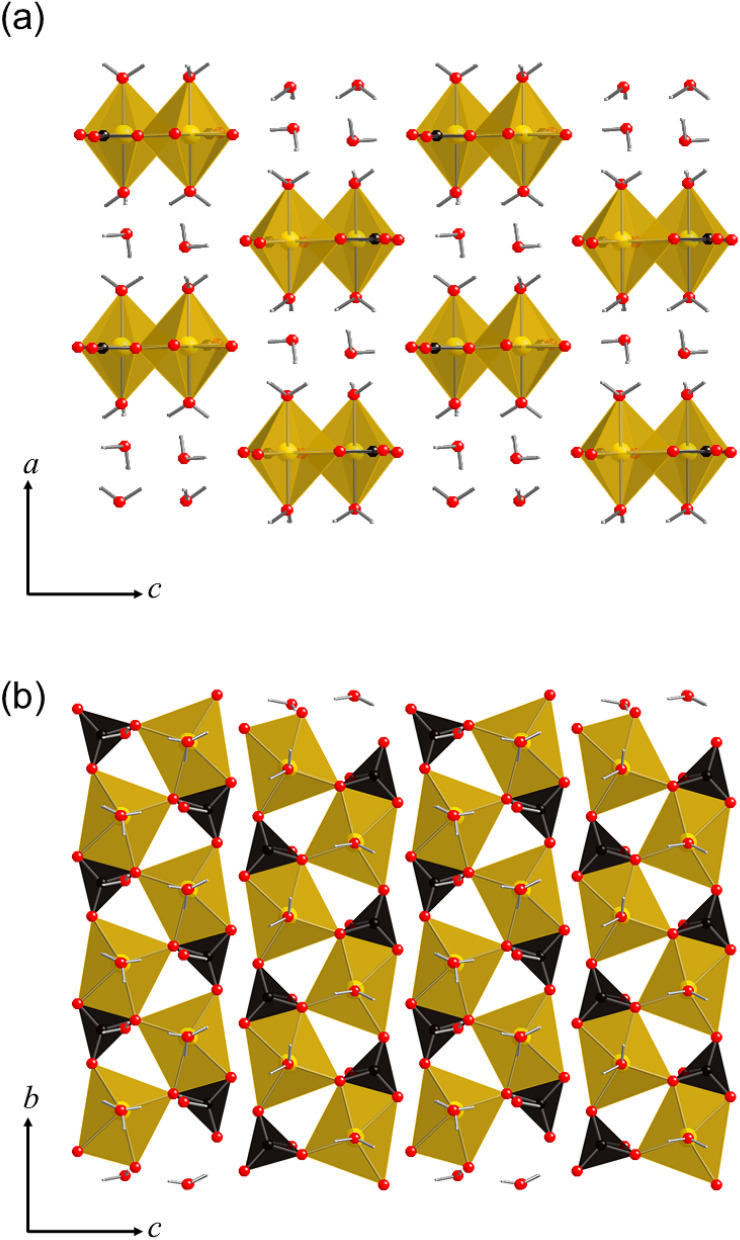
Crystal structure of nesquehonite (MgCO_3_·3H_2_O) projected along the **(a)** *b*- and **(b)** *a*-axes^9^. Yellow polyhedra and black triangles represent MgO_6_ octahedra and CO_3_ groups, respectively. Red and small white spheres denote oxygen and hydrogen atoms, respectively. The crystal structure images were generated using CrystalMaker^®^ software.

**Figure 2:**
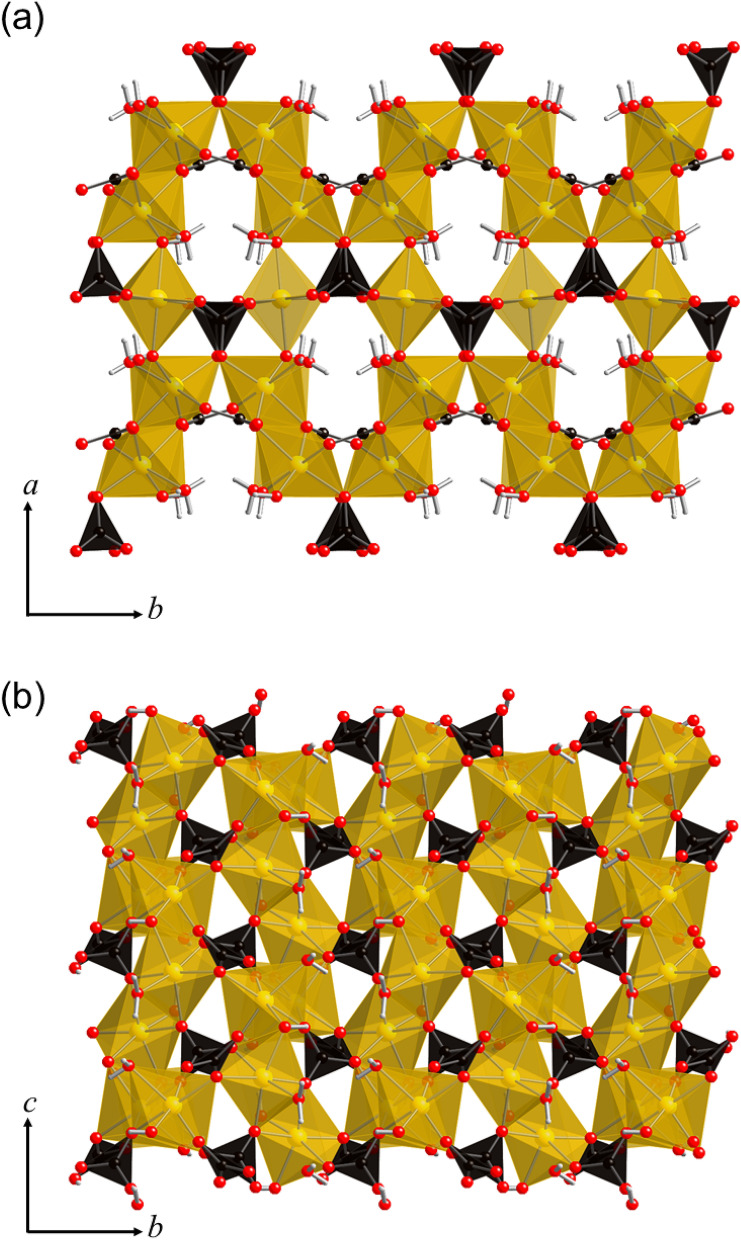
Crystal structure of hydromagnesite [Mg_5_(CO_3_)_4_(OH)_2_·4H_2_O] projected along the **(a)** *c*- and **(b)** *a*-axes^10^. Yellow polyhedra and black triangles represent MgO_6_ octahedra and CO_3_ groups, respectively. Red and small white spheres denote oxygen and hydrogen atoms, respectively. The crystal structure images were generated using CrystalMaker^®^ software.

The temperature, CO_2_ pressure, and pH all affect the type of crystalline magnesium carbonate hydrate that forms during carbonation. To date, 10 forms of crystalline magnesium carbonate hydrates have been recognized in the MgO–CO_2_–H_2_O system^8^. Nesquehonite (MgCO_3_·3H_2_O or Mg(HCO_3_)(OH)·2H_2_O), hydromagnesite [Mg_5_(CO_3_)_4_(OH)_2_·4H_2_O], and dypingite [Mg_5_(CO_3_)_4_(OH)_2_·5H_2_O] are the most dominant phases among the crystalline magnesium carbonate hydrates. The crystal structures of nesquehonite and hydromagnesite are shown in Figs. 1 and 2, respectively. Nesquehonite (Fig. 1) is composed of infinite ribbons of MgO_6_ octahedra running along the *b*-axis^8,9^. Each MgO_6_ octahedra is linked to three CO_3_ groups lying parallel to the *c*-axis. The ribbons are highly interconnected by a hydrogen-bonding network of water molecules. In contrast, hydromagnesite (Fig. 2) has a complex structure in which sheets of MgO_6_ octahedra and CO_3_ groups are sandwiched between corrugated layers of MgO_6_ octahedra and CO_3_ groups^10,11^. No hydrogen-bonding network is formed in the hydromagnesite structure. The crystal structure of dypingite is still undetermined due to a lack of single-crystal specimens suitable for structural analysis, but it is thought to be similar to that of hydromagnesite^12,13^.

Amorphous magnesium carbonate (AMC), which can be produced by mixing aqueous solutions containing Mg^2+^ and CO_3_^2−^ ions^14^, is a precursor of these crystalline magnesium carbonate hydrate materials. At lower temperatures (below approximately 55 °C), AMC crystallizes into nesquehonite by vigorous stirring in an aqueous solution^15,16^, while at higher temperatures (above approximately 55 °C), hydromagnesite forms^16–18^. Although both nesquehonite and hydromagnesite grow directly from AMC at different temperatures, the structure of AMC has not been extensively investigated. In particular, it is unclear whether AMC itself has a temperature-dependent structure (e.g., a low-temperature nesquehonite-like structure and high-temperature hydromagnesite-like structure).

In this study, we investigated the temperature dependence of the AMC structure using synchrotron X-ray scattering experiments with pair distribution function (PDF) analysis and X-ray absorption fine structure (XAFS) measurements. Herein, we report the temperature dependence of the short- and medium-range structural features of AMC.

## Results and discussion

AMC samples were synthesized at temperatures of 20, 60, and 80 °C (denoted as AMC20, AMC60, and AMC80, respectively). The water content in the AMC samples was determined using thermogravimetry/differential thermal analysis (TG/DTA). The weight losses associated with dehydration were approximately 32, 29, and 26 wt% for AMC20, AMC60, and AMC80, respectively. Therefore, the water content of the AMC samples tended to decrease as the synthesis temperature increased. The water content corresponds to the *n* value in the AMC chemical formula MgCO_3_·*x*H_2_O. From the weight loss results, it was calculated that AMC20, AMC60, and AMC80 had *x* values of 2.2, 1.9, and 1.6, respectively, suggesting that the average chemical formula of the AMC samples is approximately MgCO_3_·2H_2_O, which is in good agreement with that of AMC obtained at 15°C^14^. As mentioned below, since there are no substantial structural differences in AMC samples, the variation of water content does not affect the medium-range structural order of AMC.

### Medium-range structural order of AMC

**Figure 3:**
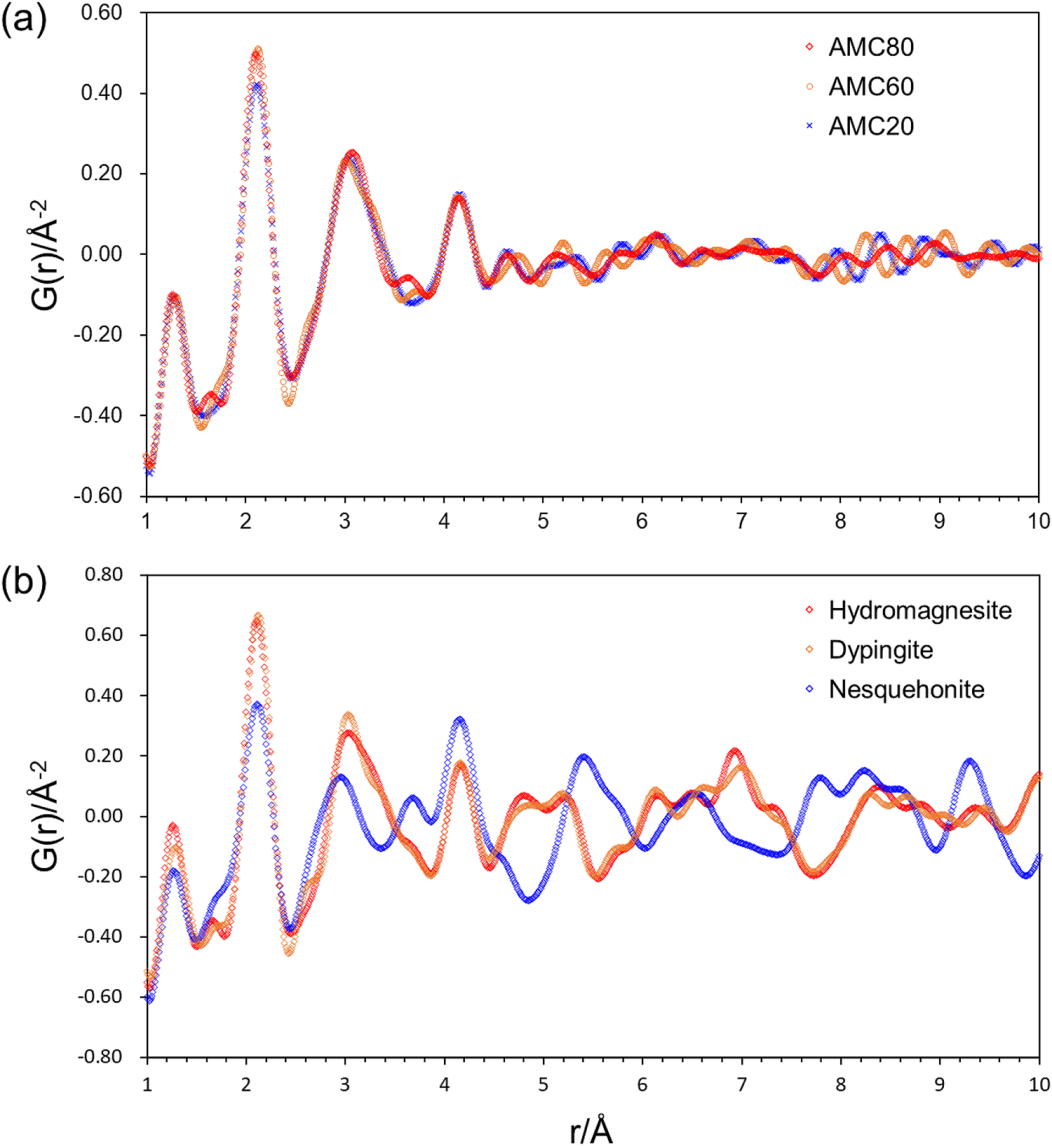
PDF patterns of different crystal structures. **(a)** AMC samples obtained at 20, 60, and 80 °C (denoted as AMC20, AMC60, and AMC80, respectively). **(b)** Nesquehonite, dypingite, and hydromagnesite crystalline magnesium carbonate hydrates obtained by stirring AMC suspensions for 2 h at 20, 60, and 80 °C, respectively.

The X-ray PDF patterns of the AMC20, AMC60, and AMC80 samples are shown in Fig. 3a. The atom–atom correlations are considerably diminished above 5 Å, which indicates that the AMC samples are fully amorphous. The PDF patterns are consistent with previous reports^19–21^. Notably, there are no substantial structural differences in the nearest-neighbor correlations among the three AMC samples, that is, the medium-range structural order of AMC exhibits no temperature dependence in this temperature range. In other words, although the crystal structure of the crystalline magnesium carbonate hydrate produced from AMC changes with temperature (from nesquehonite to hydromagnesite via dypingite), the medium-range structural order of AMC remains unchanged. AMC acts as a pluripotent source for different magnesium carbonate hydrates.

**Figure 4:**
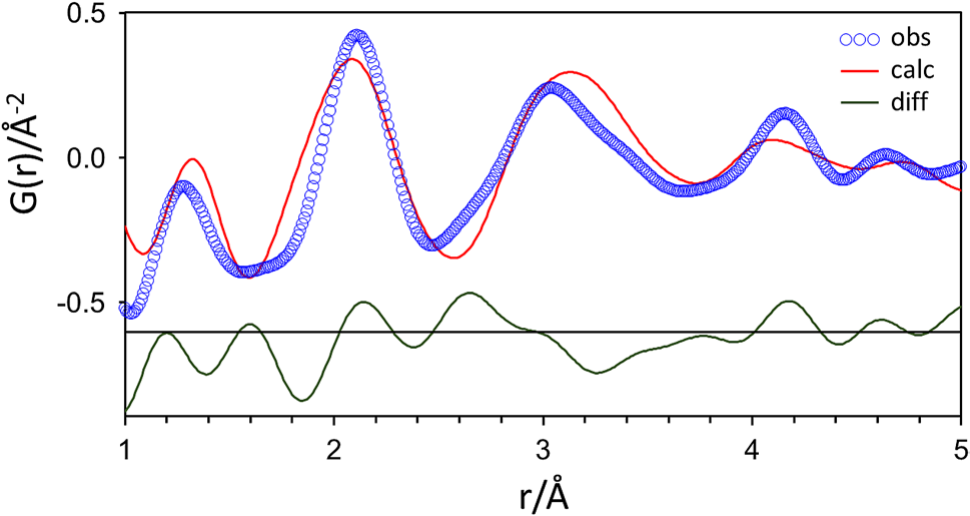
PDF fit of AMC20 between 1 and 5 Å with hydromagnesite structure^10^.

The PDF patterns of nesquehonite, dypingite, and hydromagnesite formed by stirring the AMC samples for 2 h at 20, 60, and 80 °C, respectively, are shown in Fig. 3b. The PDF pattern of hydromagnesite was very similar to the PDF patterns of all three AMCs (Fig. 3a). Figure 4 shows a PDF pattern of AMC20 fitted with the hydromagnesite structure^10^, demonstrating the close match between AMC20 and hydromagnesite. This indicates that the AMC samples have a similar medium-range order to that of hydromagnesite. The PDF pattern of hydromagnesite has been well characterized based on the crystal structure^8^. The interatomic correlation peaks at 1.3, 2.1, and 3.1 Å are assigned to C–O bonds, Mg–O bonds, and Mg–Mg interactions, respectively. The peak at 4.2 Å corresponds to the second nearest-neighbor interaction between Mg and O atoms.

The fact that the medium-range order of AMC resembles that of hydromagnesite, even when formed at 20 °C, raises the question of why the structure of AMC20 is similar to that of hydromagnesite (grown at 80 °C) instead of that of nesquehonite (grown at 20 °C). The answer may lie in the transformation behavior of nesquehonite. AMC crystallizes into nesquehonite when stirred at room temperature (below approximately 55 °C), but after a few hours to a few days, it transforms to dypingite^14,22^. This transformation is considered to occur via a solvent-mediated transformation mechanism^14^, that is, Mg^2+^ and CO_3_^2−^ ions rapidly aggregate to form AMC with the medium-range order of hydromagnesite. By stirring the AMC in aqueous solution, it immediately hydrates and transforms to the long-range order of nesquehonite. The agitated solution environment has the advantage of forming a three-dimensional hydrogen-bonding network^9^. Thus, AMC with a hydromagnesite-like structure may transform into nesquehonite by hydration and crystallization processes. Considering that all the AMC samples have a hydromagnesite-like structure, the hypothesis that nesquehonite subsequently dissolves to precipitate dypingite via a solvent-mediated transformation is quite reasonable. That is, when nesquehonite dissolves in aqueous solution and returns to AMC, its structure reverts to hydromagnesite-type structure. The transformation to dypingite is therefore very advantageous because dypingite possesses a hydromagnesite-type structure as well^12,13^.

### Short-range order of AMC

Mg K-edge X-ray absorption near-edge structure (XANES) spectroscopy is strongly dependent on the local electronegativity and multiple scattering^23^, which means that it can differentiate between structures with subtle modifications in local coordination and electronegativity^24^. This sensitivity makes it extremely useful for fingerprinting Mg-bearing phases^24^. The Mg K-edge XANES spectra of carbonates show prominent post-edge features between 1321 and 1350 eV^24^. Dolomite [MgCa(CO_3_)_2_] and calcite (CaCO_3_) share a post-edge feature at 1324 eV, but magnesite (MgCO_3_) has no such features, despite being an isostructure of dolomite and calcite, because of subtle differences in the local coordination and electronegativity.

**Figure 5:**
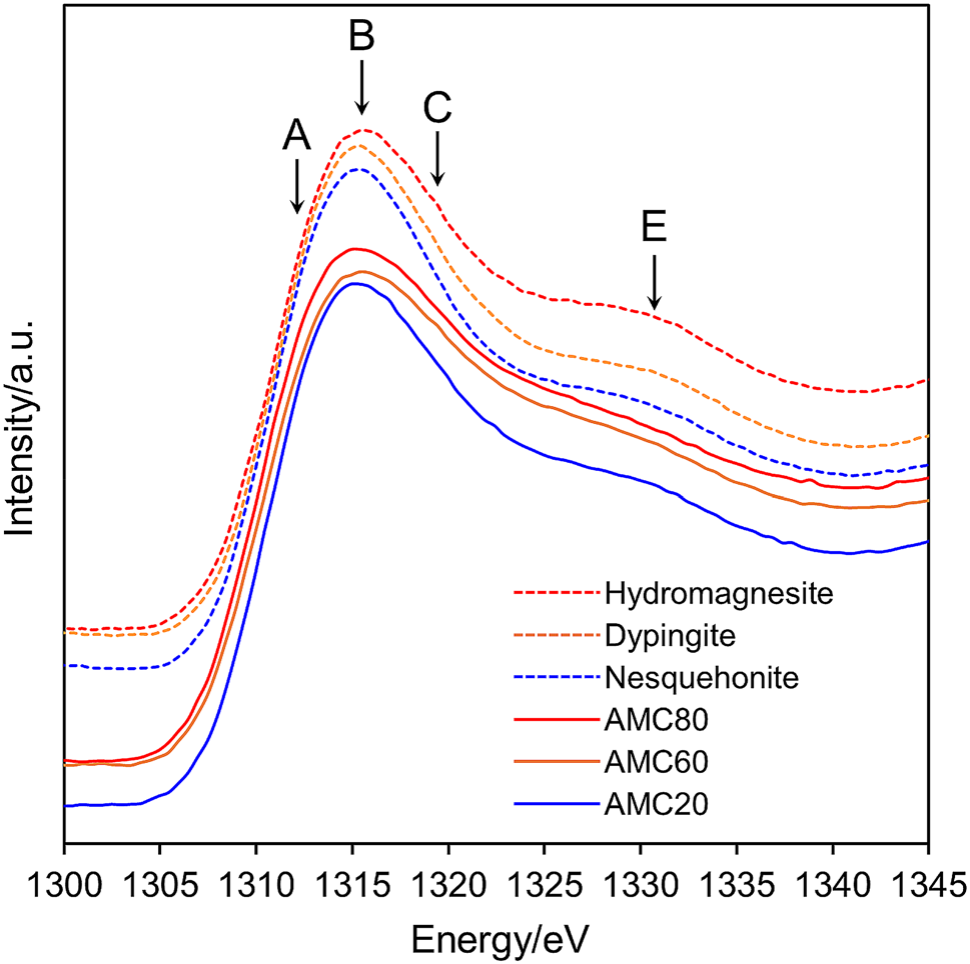
Mg K-edge XANES spectra of AMC samples obtained at 20, 60, and 80 °C (denoted as AMC20, AMC60, and AMC80, respectively) and nesquehonite, dypingite, and hydromagnesite crystalline magnesium carbonate hydrates obtained by stirring AMC suspensions for 2 h at 20, 60, and 80 °C, respectively. The letters A to E denote each peak in the paper by Finch and Allison^24^.

The Mg K-edge XANES spectra of the AMC samples are shown in Fig. 5. All three spectra have similar overall shapes in the pre- and post-edge regions. Previous studies have revealed that the near-edge region between 1306 and 1321 eV can be divided into three main components (assigned to A, B, and C)^24–27^. The near-edge region contributed by the three components showed a main peak centered at 1315 eV (marked B) with shoulders at 1312 and 1320 eV (marked A and C, respectively) (Fig. 5). A broad post-edge peak at 1330 eV (marked E) was also observed. Notably, shoulder C was more intense for AMC60 and AMC80 than for AMC20. A similar trend was observed for the crystalline phase, that is, shoulder C was stronger for hydromagnesite and dypingite than for nesquehonite. Therefore, it can be considered that the short-range order (coordination geometry) around the Mg atoms in AMC20 is highly similar to that in nesquehonite. In contrast, the Mg atoms in AMC60 and AMC80 have an identical coordination environment to those in hydromagnesite and dypingite.

The total energies, polyhedral parameters, and distortion indexes of the MgO_6_ octahedra are summarized in Table 1. In both hydromagnesite and nesquehonite, Mg ions are coordinated by six oxygen atoms to form MgO_6_ octahedra. Assuming that all the vertices are oxygen atoms, the MgO_6_ octahedra in nesquehonite have a slightly lower total energy than those in hydromagnesite. In addition, the distortion parameters^28^ of the MgO_6_ octahedra in nesquehonite are larger than those in hydromagnesite. These slight differences in the electronic structure and coordination geometry of the Mg site contributed to the observed XANES spectra.

**Table 1:** Total energy, polyhedral parameters, and distortion indexes of the MgO_6_ octahedra in hydromagnesite and nesquehonite.

Mineral name	Hydromagnesite			Nesquehonite
Site	Mg1	Mg11	Mg2	Mg
Total energy (Hartree)	− 653.087	− 653.087	− 653.082	− 653.130
Polyhedral volume (Å^3^)	11.838	11.953	11.373	11.408
Average Mg–O bond length (Å)	2.0858	2.0964	2.0484	2.0765
Quadratic elongation 〈λ_oct_〉	1.0149	1.0189	1.0052	1.0313
Bond angle variance σ_θ(oct)_^2^	50.8	65.23	17.86	104.48

The distortion parameters defined by Robinson et al.^28^ are as follows:

Quadratic elongation 〈λ_oct_〉 = $\sum_{i=1}^{6}\frac{{\left({l}_{i}/{l}_{0}\right)}^{2}}{6}$, where *l*_*i*_ is the bond length; *l*_*0*_ is the center-to-vertex distance for an octahedron.

Bond angle variance σ_θ(oct)_^2^ = $\sum_{i=1}^{12}\frac{{\left({\theta }_{i}-90^\circ \right)}^{2}}{11}$, where *θ*_*i*_ is the O–Mg–O angle.

Consequently, the short-range order (coordination environment) around the Mg atoms is slightly modified depending on the synthesis temperature, but the medium-range order of AMC remains unchanged in the temperature range from 20 to 80 °C. AMC20 therefore has a hybrid structure, featuring structural characteristics of both nesquehonite and hydromagnesite. Under an agitated solution environment at room temperature, AMC crystallizes with a nesquehonite structure by forming a hydrogen-bonding network, but at elevated temperatures, it crystallizes into dypingite or hydromagnesite without any change in the short- or medium-range order.

One of the major risks related to geological CO_2_ sequestration is the leakage of CO_2_ from the storage reservoir. In this study, it was revealed that the medium-range structural order of AMC is similar to that of hydromagnesite. AMC crystallizes into nesquehonite with stirring, and then gradually transforms to dypingite, which has a hydromagnesite-like structure. Since AMC and dypingite both have hydromagnesite-like structures^12,13^, this suggests that the hydromagnesite-like structure is the most stable of the magnesium carbonate hydrate structures. The present and previous studies allow the prediction of the long-term stability of AMC and dypingite (hydromagnesite) in aqueous conditions^14,22^. Carbonation using AMC is likely to be the most effective sequestration technique for reducing atmospheric CO_2_ concentrations.

## Experimental procedure

### Sample preparation

Commercially available MgCl_2_ (Wako Pure Chemicals Co., Inc.; ≥ 97.0% purity) and Na_2_CO_3_ (Wako Pure Chemicals Co., Inc.; ≥ 99.5% purity) were used as the starting materials. Solutions of 0.5 M MgCl_2_ and 0.5 M Na_2_CO_3_ were held at 20, 60, or 80 °C, and then mixed at a 1:1 volume ratio. The pH values of the solutions were about 10.5 without adjustment. A white suspension of AMC formed on mixing, which was immediately filtered out and washed with distilled water heated to the same temperature, and then collected and dried overnight in air. The AMC samples obtained at 20, 60, and 80 °C were denoted as AMC20, AMC60, and AMC80, respectively. X-ray diffraction patterns for AMC20, AMC60, and AMC80 are shown in Supplementary Fig. S1. Crystalline magnesium carbonate hydrate samples were prepared by the same method, except the white suspension was stirred at about 1000 rpm for 2 h after mixing. The X-ray diffraction patterns for crystalline magnesium carbonate hydrates are given in Supplementary Fig. S2. The samples prepared at 20, 60, and 80 °C with stirring for 2 h correspond to nesquehonite, dypingite, and hydromagnesite, respectively.

### Thermal analysis

The water content in the AMC samples was determined using TG/DTA (TG/DTA-7300, Seiko Instruments Inc. Japan). Approximately 10 mg of AMC and an α-alumina reference were placed in Al pans, and subsequently heated from 50 to 550 °C at a heating rate of 10 °C min^−1^ under argon flow (200 mL min^−1^).

### PDF analysis

Synchrotron X-ray total scattering measurements were performed at beamline BL22XU of SPring-8, Japan. Approximately 50 mg of the sample was loaded into a Kapton capillary tube with an inner diameter of 2 mm and length of 15 mm. The incident beam was monochromatized to a wavelength of 0.206225 Å using a Si(111) double-crystal monochromator. Data were collected in the *Q* range of 0.3 to 25 Å^−1^. The obtained X-ray total scattering data were transformed to total scattering structure functions [*S*(*Q*)] and atomic PDFs [*G*(*r*)] using PDFgetX2 software^29^. The obtained PDF profiles were analyzed using PDFgui software^30^.

### XAFS analysis

Mg K-edge XAFS measurements were performed at beamline BL11A^31^ of the Photon Factory (PF), High Energy Accelerator Research Organization (KEK), Japan. Powder samples were mounted on carbon adhesive tape in a sample holder and placed in the experimental chamber under ultra-high vacuum. A grazing incidence monochromator served as the Mg K absorption edge in the energy region from 1290 to 1350 eV. The samples were placed with their surface perpendicular to the incident X-ray beam. The spot size of the incident X-ray beam was approximately 2.0 × 0.5 mm. Energy calibration was performed using the MgCO_3_ spectrum.

### Quantum chemical calculation

Ab initio calculations were performed using the quantum chemical calculation software package Gaussian-09^32^. Nesquehonite and hydromagnesite possess MgO_6_ octahedra as interior structural units. Thus, the total energies of the MgO_6_ octahedra were calculated using the second-order Møller–Plesset (MP2) perturbation theory with the 6–311+G(d,p) basis set. The simulated structural model was built based on previous reports^9,10^. One terminal hydrogen atom was added to each oxygen atom of the MgO_6_ octahedra.

## Supplementary Information

Supplementary Figures.
