# Supplementary material for: Temperature dependence of amorphous magnesium carbonate structure studied by PDF and XAFS analyses
Source: Sci Rep. 2021 Nov 24;11:22876. doi: 10.1038/s41598-021-02261-8 (PMC8613255; doi:10.1038/s41598-021-02261-8)
Supplement: Supplementary file 1 — Supplementary Figures. [file 41598_2021_2261_MOESM1_ESM.docx]

**Supplementary Information for**

**Temperature dependence of amorphous magnesium carbonate structure studied by PDF and XAFS analyses**

Gen-ichiro Yamamoto, Atsushi Kyono^*^, Satoru Okada

Division of Earth Evolution Sciences, Graduate School of Life and Environmental Sciences, University of Tsukuba, 1-1-1 Tennodai, Tsukuba 305-8572, Japan

* Corresponding author. E-mail: kyono@geol.tsukuba.ac.jp (A. Kyono)


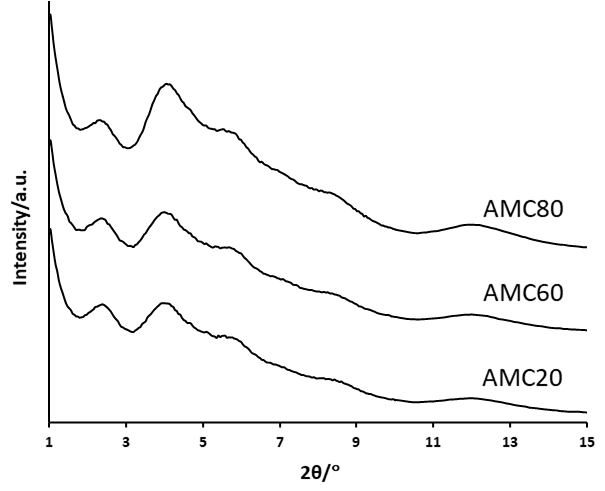


Supplementary Figure S1. Synchrotron X-ray diffraction patterns for amorphous magnesium carbonates prepared at 20 °C (AMC20), 60 °C (AMC60), and 80 °C (AMC80). The measurements were performed at beamline BL22XU of SPring-8, Japan. The X-ray wavelength was 0.206225 Å.


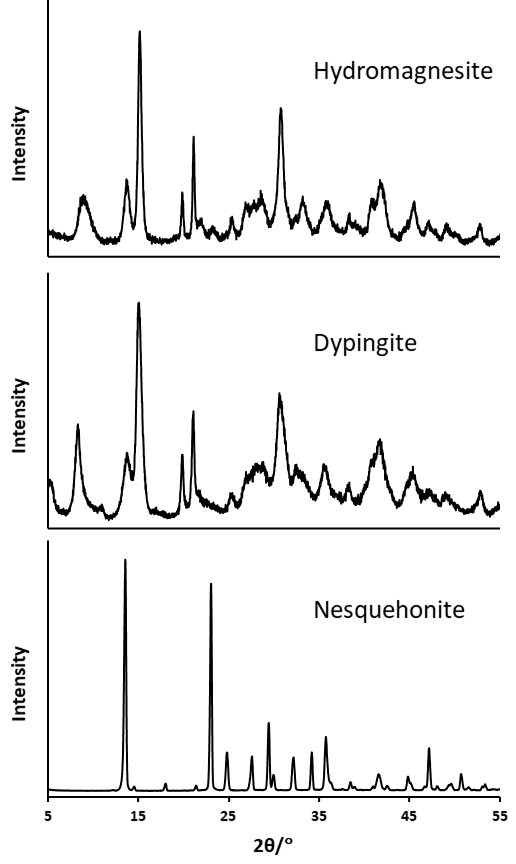


Supplementary Figure S2. X-ray diffraction patterns of crystalline magnesium carbonate hydrates prepared at 20, 60, and 80 °C. The results show that the nesquehonite, dypingite, and hydromagnesite samples each comprised a single phase.
